# Hypertensive disorders during pregnancy as a major cause of preterm birth and adverse perinatal outcomes: findings from a Brazilian National Survey

**DOI:** 10.31744/einstein_journal/2024AO0514

**Published:** 2024-05-06

**Authors:** José Paulo de Siqueira Guida, Tábata Zumpano Dias, Giuliane Jesus Lajos, Marcelo Luis Nomura, Rodolfo de Carvalho Pacagnella, Ricardo Porto Tedesco, Patricia Moretti Rehder, Samira Haddad, Maria Helena Sousa, Renato Passini, José Guilherme Cecatti, Maria Laura Costa

**Affiliations:** 1 Universidade Estadual de Campinas Campinas SP Brazil Universidade Estadual de Campinas, Campinas, SP, Brazil.; 2 Faculdade de Medicina de Jundiaí Jundiaí SP Brazil Faculdade de Medicina de Jundiaí, Jundiaí, SP, Brazil.

**Keywords:** Premature birth, Hypertension, Hypertension, pregnancy-induced, Infant, premature, Pre-eclampsia, Pregnancy complications, cardiovascular

## Abstract

**Objective:**

This study aimed to evaluate the prevalence of hypertensive disorders during pregnancy among Brazilian women with preterm births and to compare the epidemiological characteristics and perinatal outcomes among preterm births of women with and without hypertension.

**Methods:**

This was a secondary cross-sectional analysis of the Brazilian Multicenter Study on Preterm Birth. During the study period, all women with preterm births were included and further split into two groups according to the occurrence of any hypertensive disorder during pregnancy. Prevalence ratios were calculated for each variable. Maternal characteristics, prenatal care, and gestational and perinatal outcomes were compared between the two groups using χ^2^ and *t*-tests.

**Results:**

A total of 4,150 women with preterm births were included, and 1,169 (28.2%) were identified as having hypertensive disorders. Advanced maternal age (prevalence ratio (PR) 2.49) and obesity (PR= 2.64) were more common in the hypertensive group. The gestational outcomes were worse in women with hypertension. Early preterm births were also more frequent in women with hypertension.

**Conclusion:**

Hypertensive disorders of pregnancy were frequent among women with preterm births, and provider-initiated preterm births were the leading causes of premature births in this group. The factors significantly associated with hypertensive disorders among women with preterm births were obesity, excessive weight gain, and higher maternal age.

## In Brief

Hypertension during pregnancy affects one-third of preterm deliveries in Brazil, especially in non-white and older women. Women who gain excessive weight during pregnancy are more likely to develop this condition, which is associated with early preterm birth (<34 weeks) through cesarean section because of worsening maternal clinical conditions.

## Highlights

Pregnant women with increased weight gain must be surveilled for hypertension.

Non-white and older women are more likely to develop hypertension.

Maternal conditions determine most cases of preterm delivery among women with hypertension.

## Graphical abstract

**Figure:**
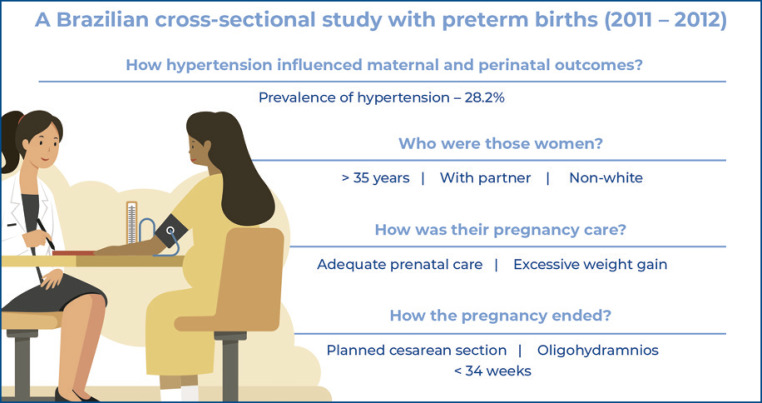

## INTRODUCTION

Chronic hypertension, gestational hypertension, preeclampsia, superimposed preeclampsia, and eclampsia are the most recognized hypertensive disorders that occur during pregnancy.^(1)^ These disorders are important in the maternal mortality and morbidity spectrum, particularly in low- and middle-income countries.^(2)^ Arterial hypertension during pregnancy is identified when two or more measures of systolic blood pressure are ≥140mmHg and/or the diastolic blood pressure is ≥90mmHg.^(3)^

Chronic hypertension is that occurring before pregnancy or before 20 weeks of gestation. When hypertension is identified after 20 weeks, it is classified as gestational hypertension, and, if accompanied by significant proteinuria or clinical or laboratory signs of organ damage, preeclampsia is diagnosed. Superimposed preeclampsia occurs in women with underlying chronic hypertension and presents as worsening of blood pressure control associated with evidence of organ damage.^(4)^

Preterm birth (PTB) is a cause of infant morbidity and mortality and is strongly associated with gestational age at delivery.^(5)^ There is inconclusive evidence about the current trends in PTB; whereas some studies report an increased rate of prematurity, ranging from 3.4 to 15%,^(6)^ other studies report a consistent reduction in PTB rates,^(7)^ probably showing disparities associated with different levels of development among countries. Preterm birth occurs due to spontaneous preterm labor or preterm premature rupture of membranes, or is medically indicated because of adverse maternal or fetal conditions. Pregnant hypertension is strongly associated with PTB.^(8)^

## OBJECTIVE

This study aimed to evaluate the prevalence of hypertensive disorders during pregnancy among Brazilian women with preterm deliveries. We also aimed to compare epidemiological characteristics and perinatal outcomes among preterm births of women with and without hypertension.

## METHODS

This is a secondary analysis of the Brazilian Multicenter Study on Preterm Birth (EMIP - *Estudo Multicêntrico de Investigação em Prematuridade*). The primary results and research protocols of this cross-sectional multicenter study have been previously published.^(9,10)^ The Brazilian Multicenter Study on Preterm Birth gathered data from all PTB cases in 20 public maternity hospitals in three Brazilian regions from April 2011 to July 2012. Informed consent was obtained after ethical approval of the original study by the Institutional Review Board of the *Universidade Estadual de Campinas* (protocol # 704/2009) and from all other participating centers.

Interviews were conducted with the included women, their medical charts were reviewed, and data were stored on a web-based electronic platform hosted by the coordinating center. Data on maternal characteristics, clinical follow-ups during pregnancy, gestational characteristics, and perinatal outcomes were also retrieved.

For this analysis, all women who delivered before 37 weeks of gestation were included, irrespective of the cause of the preterm birth, and were further divided into two groups according to the reported occurrence of hypertensive disorders of pregnancy. Women who self-reported chronic hypertension, preeclampsia, or gestational hypertension in the current pregnancy; had these diagnoses retrieved from medical charts; or had a medically indicated PTB due to hypertension, were included in the hypertension group; those who did not report any of those conditions and did not have the diagnosis reported as a cause of PTB were classified in the non-hypertension group.

Epidemiological characteristics, pregnancy outcomes, and perinatal outcomes were included in our analysis. Epidemiological characteristics included maternal factors such as age, skin color, marital status, schooling, history of diabetes, previous PTB, and previous low birth weight. Prenatal care (onset and total number of visits), weight (initial and final), substance consumption (alcohol and tobacco), anemia, and infections during pregnancy (vulvovaginitis, urinary tract infection, dental inflammation, or any other reported infection) were also used to assess the epidemiological characteristics of the population included in our sample. Gestational outcomes included fetal morbidity, onset of labor, route of delivery, gestational age at delivery, use of antenatal corticosteroids, and PTB classification. Finally, we described perinatal outcomes considering birthweight, 5^th^ minute Apgar score, ventilatory support, and length of hospital and neonatal intensive care unit (NICU) stays. We also assessed the occurrence of neonatal infections (sepsis, pneumonia, and necrotizing enterocolitis) and intraventricular hemorrhage.

A statistical analysis was performed using the SAS System for Windows (Statistical Analysis System) 9.4 software. Frequencies were obtained from each variable in each group; categorical variables were compared using the χ^2^ test. Statistical significance was set at p<0.05. The crude prevalence ratio (PR) and 95% confidence interval (95%CI) were obtained for each assessed variable. Cox regression was performed to estimate the independent association of multiple variables with two outcomes: the occurrence of hypertension during pregnancy and neonatal morbidity, and their adjusted PR with 95%CI. Variables were selected using a stepwise process, and associations were considered significant if the p value was <0.05.

## RESULTS

A total of 4,150 women with PTB were included in this analysis; of these, 1,169 (28.2%) were included in the hypertension group and 2,981 (71.8%) were included in the non-hypertension group. Among the women included in the study, 2,680 (64.6%) were 20-34 years, and 2,317 (55.8%) were non-white. Only 340 participants (8.2%) held university degrees.

Table 1 compares the maternal characteristics of both groups. Women with PTB and hypertension were older (maternal age ≥ 35 years, 21.8% *versus* 11.7%, p<0.01), and the majority had partners (80.2% *versus* 75.7%, p=0.002). They also had higher levels of education (> 12 years of education: 10.1% *versus* 7.6%, p=0.012). The reported frequency of diabetes before pregnancy was very low in both groups but was more frequent among cases of hypertension (3.8% *versus* 1.8%, p<0.01). The number of previous PTB was higher in women with hypertension (13.9% *versus* 6.5%; p<0.01). Skin color and previously low birth weight were similar in both groups.

**Table 1:** Maternal sociodemographic characteristics and clinical and obstetric background of women with preterm birth according to the diagnosis of hypertension

Maternal characteristics	Preterm births			
	Hypertension n (%), (n=1,169)	Non-hypertension n (%), (n=2,981)	p value	PR (95%CI)
Maternal age (years)			<0.001	
≤19	146 (12.5)	718 (24.1)		Ref
20–34	767 (65.7)	1,913 (64.2)		1.69 (1.42–2.02)
≥35	255 (21.8)	350 (11.7)		2.49 (2.03–3.06)
Skin color			0.203	
White	498 (42.6)	1,335 (44.8)		Ref
Other	671 (57.4)	1,646 (55.2)		1.066 (0.949–1.197)
Marital status			0.002	
With partner	937 (80.2)	2,258 (75.7)		1.207 (1.046–1.394)
No partner	232 (19.8)	723 (24.3)		Ref
Schooling (years)			0.012	
≤ 8	432 (37.6)	1,209 (41.2)		Ref
9–12	600 (52.3)	1,505 (51.2)		1.083 (0.957–1.225)
>12	116 (10.1)	224 (7.6)		1.296 (1.056–1.591)
Diabetes prior to pregnancy			<0.01	
Yes	44 (3.8)	55 (1.8)		1.601 (1.185–2.164)
No	1,124 (96.2)	2,925 (98.2)		Ref
Previous preterm birth			<0.001	
Yes	160 (13.9)	192 (6.5)		1.719 (1.454–2.031)
No	995 (86.1)	2,767 (93.5)		Ref
Previous low birthweight			0.0406	
Yes	217 (18.7)	475 (16.1)		1.139 (0.983–1.321)
No	942 (81.3)	2,481 (83.9)		Ref

PR: prevalence ratio.

Pregnancy outcomes were significantly different between the groups (Table 2). Women with hypertension had slightly higher access to prenatal care (98.0% *versus* 95.7%, p<0.01) and had a higher number of prenatal visits (61.6% *versus* 55.5%, p=0.001) compared to those with no hypertension. However, the onset of prenatal care was similar in both the groups. The frequency of obesity at the beginning and end of prenatal care was higher among women with hypertension, and this group also had a higher incidence of inadequate weight gain (43.3% *versus* 28.1%, p<0.001). Obese women had a 2.6 times higher likelihood of developing hypertension.

**Table 2:** Gestational characteristics of women with preterm birth according to the diagnosis of hypertension

Gestational characteristics	Preterm births			
	Hypertension n (%), (n=1,169)	Non-hypertension n (%), (n=2,981)	p value	PR (95%CI)
Prenatal care			<0.01	
Yes	1,146 (98.0)	2,853 (95.7)		1.881 (1.245–2.843)
No	23 (2.0)	128 (4.3)		Ref
Onset of prenatal care			0.7350	
First trimester	960 (83.8)	2,377 (83.4)		Ref
Second or third trimester	185 (16.2)	473 (16.6)		1.023 (0.874–1.197)
Number of prenatal visits			0.001	
Adequate (≥6)	599 (61.6)	1,360 (55.5)		1.198 (1.053–1.363)
Inadequate (<6)	374 (38.4)	1,091 (44.5)		Ref
Weight gain in pregnancy			<0.001	
≤7 kg	250 (24.9)	949 (37.0)		Ref
8–12 kg	319 (31.8)	893 (34.8)		1.263 (1.070–1.490)
>12 kg	434 (43.3)	721 (28.1)		1.802 (1.543–2.106)
Initial body mass index			<0.001	
<18.5kg/m^2^: underweight	35 (3.5)	267 (10.3)		0.513 (0.363–0.723)
18.5–24.99 kg/m^2^: normal	450 (45.0)	1,540 (59.5)		Ref
≥25 kg/m^2^: overweight/obesity	516 (51.5)	781 (30.2)		1.759 (1.550–1.996)
Final body mass index			<0.001	
<18.5 kg/m^2^: underweight	3 (0.3)	23 (0.9)		0.892 (0.284–2.801)
18.5–24.99 kg/m^2^: normal	128 (13.3)	861 (34.7)		Ref
≥25 kg/m^2^: overweight/obesity	828 (86.3)	1,596 (64.4)		2.639 (2.191–3.179)
Paid work in pregnancy			0.6698	
Yes	471 (88.4)	1,064 (87.6)		1.049 (0.805–1.367)
No	62 (11.6)	150 (12.4)		Ref
Smoking in pregnancy			<0.001	
Yes	115 (9.8)	475 (15.9)		Ref
No	1,054 (90.2)	2,506 (84.1)		1.519 (1.253–1.841)
Alcohol consumption in pregnancy			0.0401	
Yes (frequently)	10 (0.9)	51 (1.7)		Ref
No or seldom	1,149 (99.1)	2,911 (98.3)		1.726 (0.926–3.217)
Anemia			0.0184	
Yes	297 (26.0)	866 (29.7)		Ref
No	845 (74.0)	2,047 (70.3)		1.144 (1.002–1.306)
Vulvovaginitis in pregnancy			0.0017	
Yes	161 (23.3)	536 (29.6)		Ref
No	530 (76.7)	1,276 (70.4)		1.270 (1.065–1.515)
Urinary tract infection during pregnancy			0.477	
Yes	308 (23.9)	797 (34.2)		1.042 (0.909–1.195)
No	628 (67.1)	1,533 (65.8)		Ref
Dental inflammation/infection in pregnancy			0.3225	
Yes	209 (18.1)	497 (16.8)		1.066 (0.918–1.239)
No	944 (81.9)	2,456 (83.2)		Ref
Any other infection during pregnancy			0.859	
Yes	105 (9.1)	274 (9.3)		0.985 (0.806–1.204)
No	1,050 (90.9)	2,682 (90.7)		Ref

Smoking during pregnancy was less frequent among women with hypertension (9.8% *versus* 15.9%, p<0.001) and alcohol consumption was rare and less frequent in this group compared to those with no hypertension. Women in the hypertension group were less likely to have anemia (26.0% *versus* 29.7%, p=0.0184) compared to those with no hypertension. Urinary tract infection was the most frequent infection during pregnancy; however, the occurrence was similar between the groups, and vulvovaginitis was more frequent in women without hypertension compared to those with hypertension (Table 2).

All variables assessing pregnancy outcomes differed significantly among the groups (Table 3). Fetal morbidity was more frequent in the hypertension group (33.3% *versus* 20.8%, p<0.001) than the no hypertension group. Women with hypertension had a 6.4 higher chance of undergoing a planned cesarean section, and 82.2% of the preterm deliveries in this group were provider-initiated, compared to the no hypertension group. Amniotic fluid disorders were more frequent in the hypertensive group with marked oligohydramnios (23.1% *versus* 18.2%, p<0.01) compared to the no hypertension group. The occurrence of early PTB (<34 weeks) was more frequent in the hypertension group (41.4% *versus* 35.7%, p<0.001) compared to the no hypertension group.

**Table 3:** Pregnancy outcomes of women with preterm birth according to the diagnoses of hypertension

Pregnancy outcomes	Preterm births			PR (95%CI)
	Hypertension n (%), (n=1,169)	Non-hypertension n (%), (n=2,981)	p value	
Neonatal morbidity			<0.001	
No	733 (66.7)	2,190 (79.2)		Ref
Yes	366 (33.3)	576 (20.8)		1.550 (1.367–1.757)
Onset of labor			<0.001	
Spontaneous	198 (16.9)	2,019 (67.7)		Ref
Induction of labor	189 (16.2)	385 (12.9)		3.690 (3.023–4.503)
Planned cesarean section	782 (66.9)	577 (19.4)		6.443 (5.513–7.531)
Route of birth			<0.001	
Vaginal	222 (19.0)	1,710 (57.4)		Ref
Cesarean section	947 (81.0)	1,271 (42.6)		3.716 (3.210–4.300)
Amniotic fluid disorders			<0.01	
Oligohydramnios	253 (23.1)	496 (18.2)		1.221 (1.060–1.406)
Polyhydramnios	25 (2.3)	98 (3.6)		0.735 (0.494–1.095)
No	817 (74.6)	2,136 (78.2)		Ref
Preterm birth classification			<0.001	
Spontaneous preterm birth	208 (17.8)	2,474 (83.0)		8.441 (7.266–9.806)
Provider-initiated preterm birth	961 (82.2)	507 (17.0)		Ref
Gestational age at birth			<0.001	
<34 weeks	484 (41.4)	1,065 (35.7)		1.186 (1.056–1.333)
34–36 weeks	685 (58.6)	1,916 (64.3)		Ref

Table 4 presents perinatal outcomes in both groups. Extreme low birthweight was more frequent in the hypertension group (27.1% *versus* 18.8%, p<0.001), and those newborns demanded more ventilatory support (56.8% *versus* 49.7%, p<0.001) than the no hypertension group; however, 5^th^ minute Apgar scores ≤7 were higher in the non-hypertension group (12.1% *versus* 14.9%, p=0.02). Intubation at birth was similar in both groups. The average length of hospital stay and neonatal care unit stay were significantly higher among women with hypertension compared to those without. The frequency of sepsis, intraventricular hemorrhage, pneumonia, and oxygen therapy at 28 and 56 days of life was similar; necrotizing enterocolitis was higher in the hypertension group (3.6% *versus* 2.2%, p=0.035) than in the no hypertension group. The occurrence of newborn death was similar in both groups, with an overall frequency of 8.5%.

**Table 4:** Perinatal outcomes among women with preterm birth according to the diagnosis of hypertension

Perinatal results	Preterm births			
	Hypertension n (%), (n=1,169)	Non-hypertension n (%), (n=2,981)	p value	PR (95%CI)
Birthweight			<0.001	
≤1500 g	316 (27.1)	558 (18.8)		1.621 (1.374–1.912)
1501 to 2500 g	591 (50.7)	1,510 (50.9)		1.261 (1.089–1.460)
>2500 g and ≤4000 g	255 (21.9)	888 (30.0)		Ref
>4000 g	3 (0.3)	8 (0.3)		1.223 (0.392–3.816)
5^th^ minute Apgar score ≤7			0.02	
Yes	140 (12.1)	435 (14.9)		0.841 (0.705–1.003)
No	1,015 (87.9)	2,490 (85.1)		Ref
Orotracheal intubation at birth			0.5784	
Yes	189 (17.0)	457 (16.3)		1.039 (0.888–1.215)
No	922 (83.0)	2,350 (83.7)		Ref
Surfactant use			0.0470	
Yes	194 (17.7)	419 (15.1)		1.142 (0.978–1.334)
No	901 (82.3)	2,351 (84.9)		Ref
Fetal malformation			0.005	
Yes	96 (8.7)	328 (11.8)		Ref
No	1,006 (91.3)	2,449 (88.2)		1.286 (1.043–1.585)
Average length of hospital stay (days)	17.7±19.6	14.9±19.5	<0.001	
Average length of NICU stay (days)	11.7±19.4	9.6±16.9	<0.001	
Ventilatory support			<0.001	
Yes	632 (56.8)	1,396 (49.7)		1.229 (1.091–1.384)
No	480 (43.2)	1,413 (50.3)		Ref
Any neonatal morbidity			<0.001	
Yes	833 (74.8)	1,923 (68.6)		1.248 (1.090–1.428)
No	281 (25.2)	879 (31.4)		Ref
Sepsis			0.0687	
Yes	214 (27.0)	562 (30.5)		0.885 (0.757–1.036)
No	579 (73.0)	1280 (69.5)		Ref
Respiratory distress			0.004	
Yes	654 (79.4)	1,412 (74.2)		1.229 (1.038–1.455)
No	170 (20.6)	490 (25.8)		Ref
Intraventricular hemorrhage			0.999	
Yes	59 (9.4)	139 (9.4)		1.00 (0.765–1.307)
No	568 (90.6)	1,338 (90.6)		Ref
Necrotizing enterocolitis			0.035	
Yes	29 (3.6)	40 (2.2)		1.392 (0.961–2.016)
No	786 (96.4)	1,813 (97.8)		Ref
Pneumonia			0.7014	
Yes	47 (5.8)	114 (6.1)		0.954 (0.711–1.281)
No	770 (94.2)	1,744 (93.9)		Ref
Oxygen therapy at 28 days			0.8566	
Yes	72 (8.9)	161 (8.6)		1.019 (0.800–1.298)
No	714 (91.1)	1,702 (91.4)		Ref
Oxygen therapy at 56 days			0.7624	
Yes	24 (3.0)	59 (3.2)		0.950 (0.633–1.426)
No	776 (97.0)	1,771 (96.8)		Ref
Newborn’s condition at discharge or hospital transfer			0.0868	
Alive	1,037 (92.8)	2,581 (91.1)		Ref
Dead	81 (7.2)	253 (8.9)		0.846 (0.675–1.061)

NICU: neonatal intensive care unit.

Higher maternal age is independently associated with the occurrence of hypertension during pregnancy. Women aged 20–34 years had a 2.5 higher likelihood and those aged >35 years had a 3.4 higher chance of PTB than those aged <20 years. A weight gain > 12kg increased the risk of hypertension during pregnancy by 2.18, and obese women also had a greater chance of hypertension compared to those with a BMI <25 m/m^2^ (Table 5).

**Table 5:** Factors independently associated with hypertensive diseases of pregnancy among women with preterm birth in Brazil

Factor	p value	PR_adj_ (95CI)
Maternal age (between 20–34 years)	0.0453	2.494 (1.019–6.103)
Maternal age (>35 years)	0.0096	3.372 (1.346–8.449)
Weight gain (>12 kg)	<0.001	2.184 (1.546–3.086)
Obesity (BMI >25 kg/m^2^)	<0.001	1.753 (1.341–2.291)

BMI: body mass index.

To evaluate factors associated with neonatal morbidity, all variables of tables 1, 2, and 3 were tested; however, only delivery before 34 weeks was independently associated with this outcome (PR= 1.69, 95%CI=1.41–2.01) (data not shown).

## DISCUSSION

We aimed to compare the characteristics of PTB among women with and without hypertension using a large database of Brazilian PTB cases. More than 20% of the women had hypertensive disorders of pregnancy; advanced maternal age, higher educational levels, and having a partner were more likely to occur in the hypertension group. Infections were frequent in both groups; however, tobacco and alcohol consumption were not frequent, and smoking was significantly less common among women with hypertension. Women with hypertension had worse gestational characteristics, such as fetal morbidity, early PTB, and oligohydramnios; therefore, they received more antenatal corticosteroids than those without hypertension. Due to the earlier gestational age at birth, newborns in the hypertension group required more ventilatory support than those in the no hypertension group; however, other complications and deaths were similar. Obesity, uncontrolled weight gain during pregnancy, and advanced maternal age were associated with hypertension, whereas extreme preterm birth was associated with neonatal morbidities.

The women included in this study were mainly non-white, 20–34 years of age, and most did not have a university degree. We did not collect data on family income. However, we can infer that our sample comprised young, poor, and non-white women. The Brazilian Institute of Geography and Statistics evaluated the characteristics of the Brazilian population through a national survey called the *Programa Nacional de Avaliação de Domicílios Contínua* (PNAD *Contínua*), with the most recent report published in 2022.^(11)^ According to a survey, 55.9% of the Brazilian population is non-white, most of the female population is aged between 20–44 years, and only 19.2% have a university degree. The population included in this study was, therefore, similar to the Brazilian population, and our results probably reflect the reality of women attending public health settings, which is the majority in the country.

We showed that hypertensive disorders during pregnancy affected one-fifth of all the PTB cases included in the cohort. Chronic hypertension is the most frequent non-communicable disease in Brazil, and is more frequently self-reported by women^(12)^ while preeclampsia prevalence ranges from 4.7% to 7.5%.^(13,14)^ Our prevalence of hypertensive disorders of pregnancy is representative of the national background of a high prevalence of chronic hypertension and the high prevalence of preeclampsia in middle-income countries such as Brazil.^(15)^ Unfortunately, our study does not allow for accurate diagnosis of different hypertensive disorders because the original study was not developed specifically for the study of hypertension in pregnancy. The criteria for each hypertensive disorder of pregnancy were not obtained during data collection, and we grouped chronic hypertension, gestational hypertension, and preeclampsia for this analysis. We recognize this as an important limitation.

Another important aspect related to the higher frequency of hypertensive disorders of pregnancy compared to other previous reports was that we obtained data specifically from a cohort of women with PTB; among the 4,150 PTB included, 1,468 were provider-initiated.^(10)^ Among these women, 598 (40.73%) had hypertensive disorders during pregnancy. These two factors may explain the high frequency of hypertensive disorders during pregnancy in our study.

Clinical phenotypes related to PTB have already been explored using the EMIP database;^(16)^ our results suggest that women with hypertension who had PTB were older, had a better educational level, and had a partner, suggesting that they had a higher economic status when compared to those with no hypertension. They also attended more prenatal care, possibly because this group included women with chronic hypertension and were more aware of their condition, with increased surveillance of maternal conditions and pregnancy outcomes.

Another interesting point regarding the clinical phenotypes of women with PTB and hypertension is that most were obese at the beginning and had excessive weight gain during pregnancy; therefore, almost all were obese at the end of pregnancy. These four easily identified characteristics (age, educational level, marital status, and obesity) in women with hypertension are likely to be associated with medically indicated PTB, and healthcare providers should be aware of this association.^(16)^

Most PTB cases in the hypertensive group occurred before 34 weeks of gestation. These characteristics may explain the increased use of antenatal corticosteroids in this group. The use of corticosteroids was previously evaluated in EMIP^(17)^ and the results showed better outcomes among those who received this intervention before 34 weeks of preterm labor. In hypertensive disorders in pregnancy, mostly preeclampsia, the fetuses are exposed to oxidative stress, chronic hypoxia, and malnutrition. This hostile environment can cause intrauterine stress and fetuses can be exposed to endogenously elevated steroid levels. Therefore, the effect of antenatal corticosteroid treatment on neonatal outcomes in pregnancies complicated with hypertension is doubtful. In addition, approximately 30-50% of women with early onset hypertensive disorders have fetal growth restrictions, and data on the efficacy of antenatal corticosteroid treatment in women with growth-restricted fetuses are limited and conflicting. Despite these doubts, there is robust evidence in recent literature that a single course of corticosteroids (betamethasone or dexamethasone) reduces the risk of perinatal death and neonatal complications, including respiratory distress syndrome, necrotizing enterocolitis, and intensive care admissions in pregnancies at risk of preterm birth regardless of the cause; subgroup analyses indicate that there is no evidence to suggest any difference in the effect of preterm birth as a result of hypertensive disease.^(18-20)^

The incidence of cesarean sections was higher in the hypertension group than in the control group. There are two possible explanations for this. Many of these deliveries occurred before 34 weeks, and most women had their onset as a planned cesarean section due to concerns about the induction of labor before 32 weeks.^(20)^ The other explanation is that Brazil has one of the highest cesarean section rates worldwide,^(21)^ and hypertension was probably not the only (or even the primary) contributor to the decision to perform a cesarean section.

Newborns had similar performance in both groups for most of the considered variables; however, the results differed in the need for ventilatory support (PR= 1,229, 95%CI= 1,091–1,384), respiratory distress (PR= 1,229, 95%CI=1,038–1,455), and length of stay in the NICU and hospital. These worse results among women with hypertension are justified by the fact that deliveries at less than 34 weeks and birth weights less than 1,500g were more frequent in that group. This was probably due to the worsening of the maternal clinical conditions, which resulted in medically indicated preterm delivery. These results also reinforce the need for corticosteroids for fetal lung maturation, as advised by the International Society of Studies in Hypertension in Pregnancy.^(21)^

Interventions and outcomes during pregnancy can affect the health of women.^(22)^ Hypertensive disorders of pregnancy are major risk factors for pregnancy complications; women with these conditions are at a higher risk of stroke, coronary artery disease, cardiac arrhythmias, and chronic kidney disease during long-term follow-up.^(23)^ Knowing that 20% of women are affected by hypertensive disorders of pregnancy highlights the importance of adequate follow-up to prevent cardiovascular morbidity.

Our study has significant limitations. We only included women with PTB, and the results are not generalizable to the obstetric population. Another critical point previously discussed is the classification of women with hypertension, as we grouped women with different types of hypertensive disorders of pregnancy into the same group.

The strength of this analysis is that it presents the significant impact of hypertension among PTB cases in our setting, highlighting the importance of adequate antenatal care and follow-up to enable insufficient diagnosis and management. Early onset preeclampsia (<34 weeks) is associated with an increased risk of adverse maternal and perinatal outcomes but also an increased risk of long-term hypertension and cardiovascular disease.^(23)^

Hypertensive disorders of pregnancy are frequent among women with PTB and are associated with advanced maternal age, obesity, and uncontrolled weight gain. Women with hypertension had a higher incidence of early PTB—mostly provider-initiated—through cesarean sections. Low birth weight and need for ventilatory support were the main perinatal complications, and delivery below 34 weeks was associated with neonatal morbidity.
